# PHLDA1 modulates microglial response and NLRP3 inflammasome signaling following experimental subarachnoid hemorrhage

**DOI:** 10.3389/fimmu.2023.1105973

**Published:** 2023-02-17

**Authors:** Jinqing Lai, Genwang Chen, Zhe Wu, Shaoyang Yu, Rongfu Huang, Yile Zeng, Weibin Lin, Chunmei Fan, Xiangrong Chen

**Affiliations:** ^1^Department of Neurosurgery, The Second Affiliated Hospital of Fujian Medical University, Quanzhou, Fujian, China; ^2^Centre of Neurological and Metabolic Research, The Second Affiliated Hospital of Fujian Medical University, Quanzhou, Fujian, China; ^3^Clinical Lab and Medical Diagnostics Laboratory, The Second Affiliated Hospital of Fujian Medical University, Quanzhou, Fujian, China

**Keywords:** subarachnoid hemorrhage, neuroinflammation, microglial polarization, PHLDA, NLRP3

## Abstract

Balancing microglia M1/M2 polarization is an effective therapeutic strategy for neuroinflammation after subarachnoid hemorrhage (SAH). Pleckstrin homology-like domain family A member 1 (PHLDA1) has been demonstrated to play a crucial role in immune response. However, the function roles of PHLDA1 in neuroinflammation and microglial polarization after SAH remain unclear. In this study, SAH mouse models were assigned to treat with scramble or PHLDA1 small interfering RNAs (siRNAs). We observed that PHLDA1 was significantly increased and mainly distributed in microglia after SAH. Concomitant with PHLDA1 activation, nod-like receptor pyrin domain-containing protein 3 (NLRP3) inflammasome expression in microglia was also evidently enhanced after SAH. In addition, PHLDA1 siRNA treatment significantly reduced microglia-mediated neuroinflammation by inhibiting M1 microglia and promoting M2 microglia polarization. Meanwhile, PHLDA1 deficiency reduced neuronal apoptosis and improved neurological outcomes after SAH. Further investigation revealed that PHLDA1 blockade suppressed the NLRP3 inflammasome signaling after SAH. In contrast, NLRP3 inflammasome activator nigericin abated the beneficial effects of PHLDA1 deficiency against SAH by promoting microglial polarization to M1 phenotype. In all, we proposed that PHLDA1 blockade might ameliorate SAH-induced brain injury by balancing microglia M1/M2 polarization *via* suppression of NLRP3 inflammasome signaling. Targeting PHLDA1 might be a feasible strategy for treating SAH.

## 1 Introduction

Subarachnoid hemorrhage (SAH), a devastating acute cerebrovascular event, has a poor prognosis with a high rate of neurocognitive impairment in patients. Currently, accumulating evidence has proposed that early brain injury (EBI) may be a determinant factor for SAH-induced long-term neurocognitive sequelae (1–3). Unfortunately, no effective pharmaceutical strategy has been identified to interfere with the development of EBI. Hence, identifying new drug targets to improve SAH outcomes is urgently needed.

Neuroinflammation has been verified as a crucial contributor to EBI progression after SAH (4–6). It is known that microglia, key innate immune cells of the brain, are rapidly activated to various acute brain injuries (6–8). After activation, microglia can exhibit different phenotypes (M1 and M2 phenotypes) and exert distinct functions. M1 microglia could induce proinflammatory mediators and increase reactive oxygen species (ROS). By contrast, M2 microglia exhibit anti-inflammatory effects by secreting anti-inflammatory meditators. Interestingly, microglia can switch their phenotype under different microenvironments. In a variety of neurological disorders, suppression of microglia M1 polarization and promotion of M2 microglia could effectively reduce acute brain injuries and improve neurological outcomes (9–11). Thus, modulating microglia M1/M2 polarization might be a feasible method to mitigate EBI.

Recently, a substantial number of studies have revealed that pleckstrin homology-like domain family A member 1 (PHLDA1) plays a crucial role in oxidative stress and immunological regulation (12–15). In a model of cerebral ischemia/reperfusion injury, PHLDA1 blockade ameliorated the acute brain injury by switching microglia M1/M2 polarization *via* inhibiting nod-like receptor pyrin domain-containing protein 3 (NLRP3) inflammasome signaling (12). Another study reported that PHLDA1 deficiency mitigated motor deficits and microglia-mediated neuroinflammation in Parkinson’s disease models (13). However, the function roles of PHLDA1 in microglia-mediated immune response after SAH remain unclear. NLRP3 inflammasome has been demonstrated to implicate in neuroinflammation after SAH by modulating microglial polarization (4, 16). Notably, blockade of NLRP3 inflammasome activation could exert beneficial effects in different brain injuries (17–19). Herein, we hypothesized that PHLDA1 inhibition might mitigate neuroinflammation and the subsequent neurobehavior deficits after SAH through the NLRP3 inflammasome signaling pathway.

## 2 Material and methods

### 2.1 Establishment of SAH model

Adult male C57BL/6 mice (8-10 wk old, weighing 20–25 g) were obtained from the Animal Core Facility of Fujian Medical University. All experimental procedures were complied with the rules for animal research by Fujian Medical University. Briefly, mice were anesthetized with isoflurane. After the common, external and internal carotid arteries were exposure, a marked 6-0 filament was employed to puncture the origin of the left middle cerebral artery through the internal carotid artery (20). Animals in sham group received similar procedures without the artery puncture. The SAH severity grading score was recorded according to previous studies (21). Mice with a SAH grading score of less than 8 were excluded.

### 2.2 Study design

In the first experiment, mice were assigned to sham group (n = 6) and post-SAH (6 h, 12 h, 24 h, 48 h, 72 h) (n = 6 per group). Western blot and immunofluorescence staining were performed in the experiment. In the second experiment, mice were assigned to sham group, vehicle-treated SAH group, Scramble small interfering RNA (siRNA)-treated SAH group, and PHLDA1 siRNA-treated SAH group (n = 12 per group). Animals were sacrificed at 24 h or 72 h after SAH. Post-treatment assessments included neurobehavior tests, western blot, immunofluorescence staining, TUNEL staining, enzyme-linked immunosorbent assay (ELISA), and biochemical estimation. In the third experiment, mice were assigned to sham group, vehicle-treated SAH group, PHLDA1 siRNA-treated SAH, and PHLDA1 siRNA plus nigericin -treated SAH group (n = 12 per group). Post-treatment assessments included neurobehavior tests, western blot, immunofluorescence staining, TUNEL staining, ELISA, and biochemical estimation.

### 2.3 Drug administration

For PHLDA1 knockdown, a volume of 3μl PHLDA1 siRNA (Santa Cruz Technology) or scramble siRNA was dissolved in transfection solution and then injected into the lateral ventricles at 48 h before the construction of SAH model. Nigericin (MedChemExpress, 2 μg), a potent NLRP3 activator, was prepared in 2 μl ethanol and physiologic saline. Nigericin or vehicle was intracerebroventricularly administered at 2 h before SAH operation. The dose of nigericin and administration route were based on previous studies (22).

### 2.4 Neurobehavioral tests

The modified Garcia scale test was used to evaluate neurological deficits as previously reported (23). Six measurements were included in this score system. The higher score suggested the better neurobehavioral outcomes. For motor function, the beam-walking score test was performed according to previous reports (24). The animals’ walking distance within 1 min were recorded. Neurobehavior tests were conducted in a blinded manner.

### 2.5 ELISA

The supernatant of brain samples was collected. The levels of interleukin (IL)-1β, IL-6, IL-18, and IL-10 were detected by using commercially available kits (Multi Sciences). The detailed methods were conducted according to the manufacturer’s instructions.

### 2.6 Western blotting

The brain tissue and protein samples were prepared according to previous studies (25). Briefly, the protein samples were loaded onto SDS-PAGE gels and transferred to PVDF membranes. The membranes were blocked with 5% non-fat milk. After that, they were incubated with primary antibodies: PHLDA1 (1:1000, Abcam), NLRP3 (1:200, Santa Cruz Biotechnology), ASC (1:200, Santa Cruz Biotechnology), caspase-1 (1:200, Santa Cruz Biotechnology), cleaved caspase-1 (1:200, Santa Cruz Biotechnology), and β-actin (1:3000, Bioworld Technology) in a 4°C freezer. Then, membranes were incubated with corresponding secondary antibodies. ImageJ software was employed to measure relative intensity.

### 2.7 Immunofluorescence staining

The detailed methods were performed according to previous studies (26). In brief, the frozen tissue sections were treated with Triton X-100 (0.3%) and then blocked with 5% goat serum. After that, sections were incubated with primary antibodies: PHLDA1 (Abcam), CD16/32 (BD Biosciences), CD206 (Invitrogen), NeuN (EMD Millipore), IL-1β (Santa Cruz Biotechnology), and Iba-1 (Santa Cruz Biotechnology) in a 4°C freezer. After that, they were incubated with corresponding secondary antibodies followed by using DAPI staining. The slices were then observed under a fluorescence microscope.

### 2.8 TUNEL staining

TUNEL staining was performed by using a commercially available kit (Beyotime Biotechnology). Brain sections were incubated with primary antibody against NeuN in a 4°C freezer. After that, the sections were incubated with TUNEL reaction mixture. The slides were then washed and counterstained with DAPI. The slices were observed under a fluorescence microscope.

### 2.9 Statistical analysis

Data are expressed as mean ± SD. Statistical analysis was conducted with Graph- Pad Prism 8 software. Statistical evaluation was performed using one-way ANOVA or two-way ANOVA with Tukey’s *post hoc* test. The significant *P*-value was < 0.05.

## 3 Results

### 3.1 Time course and cellular expression of PHLDA1 and NLRP3 after SAH

**Figure 1:**
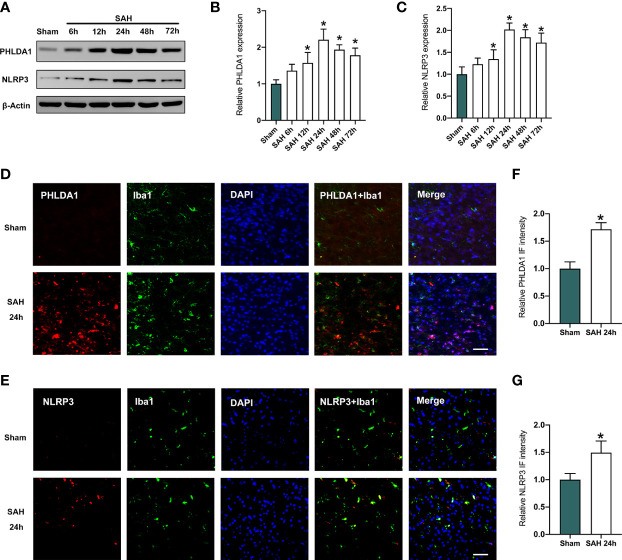
Expression levels of PHLDA1 and NLRP3 were increased after SAH. **(A)** Representative western blots for PHLDA1 and NLRP3 expressions in the early period after SAH. Western blot analysis of PHLDA1 **(B)** and NLRP3 **(C)** expressions after SAH (n = 6 per group). **(D, E)** Representative immunofluorescence images of PHLDA1 and NLRP3 co-localized with Iba1 in temporal cortex after SAH. Quantification of PHLDA1 **(F)** and NLRP3 **(G)** immunoactivities in microglia (n = 6 per group). ^*^P < 0.05. Scale bar=50 μm. Data are expressed as mean ± S.D.

Mounting evidence has indicated that PHLDA1 and NLRP3 might interact with each other. PHLDA1 activation could induce NLRP3 inflammasome signaling. In this experiment, western blot (**Figure 1A**) was performed to investigate the protein expression of PHLDA1 and NLRP3. As shown in **Figures 1B, C**, the expression of PHLDA1 and NLRP3 markedly increased in the early period after SAH, and peaked at 24 h post-SAH (*P* < 0.05). In addition, double immunofluorescence staining indicated that the enhanced PHLDA1 and NLRP3 were mainly distributed in microglia after SAH (*P* < 0.05) (**Figures 1D–G**).

### 3.2 PHLDA1 deficiency inhibited NLRP3 inflammasome signaling activation after SAH

**Figure 2:**
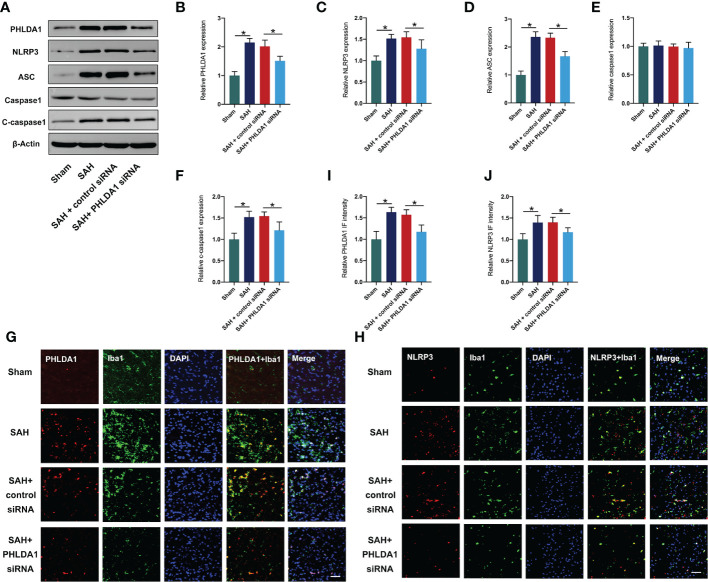
PHLDA1 deficiency suppressed NLRP3 inflammasome signaling after SAH. **(A)** Representative western blots for PHLDA1, NLRP3, ASC, Caspase1, and Cleaved caspasse1 expressions after SAH. Western blot analysis of PHLDA1 **(B)**, NLRP3 **(C)**, ASC **(D)**, caspase1 **(E)**, and cleaved caspasse1 **(F)** expressions after SAH (n = 6 per group). Representative immunofluorescence images of PHLDA1 **(G)** and NLRP3 **(H)** co-localized with Iba1 in temporal cortex. Quantification of PHLDA1 **(I)** and NLRP3 **(J)** immunoactivities in microglia (n = 6 per group). ^*^P < 0.05. Scale bar=50 μm. Data are expressed as mean ± S.D.

Previous study has demonstrated that PHLDA1 activation could induce NLRP3 inflammasome signaling. We applied PHLDA1 siRNA to inhibit PHLDA1 expression and explore whether PHLDA1 deficiency could reduce NLRP3 inflammasome activation. As shown, western blot results (**Figure 2A**) showed that PHLDA1 siRNA significantly reduced PHLDA1 expression after SAH (*P* < 0.05). Moreover, the activated NLRP3 inflammasome signaling pathway was markedly suppressed by PHLDA1 siRNA (*P* < 0.05) (**Figures 2A–F**). Consistently, double immunofluorescence staining confirmed that PHLDA1 siRNA significantly decreased PHLDA1 and NLRP3 expression in microglia in the brain cortex after SAH (*P* < 0.05) (**Figures 2G–J**).

### 3.3 PHLDA1 deficiency reduced inflammatory response

**Figure 3:**
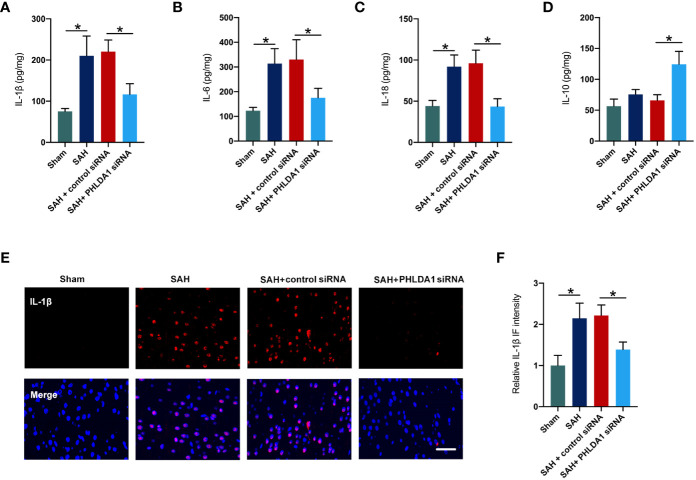
PHLDA1 deficiency mitigated inflammatory insults after SAH. ELISA analysis of IL-1β **(A)**, IL-6 **(B)**, IL-18 **(C)**, and IL-10 **(D)** expressions (n = 6 per group). **(E)** Representative immunofluorescence images of IL-1β staining in temporal cortex. **(F)** Quantification of IL-1β immunoactivities (n = 6 per group). ^*^P < 0.05. Scale bar=50 μm. Data are expressed as mean ± S.D.

The anti-inflammatory effects of PHLDA1 blockade have been verified in other diseases models. We further investigated the influence of PHLDA1 deficiency on inflammatory response after SAH. By using ELISA kits, we found that SAH insults induced a significant increase in proinflammatory cytokines release, including IL-1β, IL-6, and IL-18 (*P* < 0.05) (**Figures 3A–C**). All these cytokines were decreased by PHLDA1 deficiency (*P* < 0.05). In addition, PHLDA1 deficiency significantly induced an increase in IL-10 expression after SAH (*P* < 0.05) (**Figure 3D**). Simultaneously, IL-1β immunofluorescence staining verified that PHLDA1 deficiency significantly decreased the enhanced levels of IL-1β in the brain cortex after SAH (*P* < 0.05) (**Figures 3E, F**).

### 3.4 PHLDA1 deficiency promoted M2 microglia polarization and prevented M1 microglia polarization after SAH

**Figure 4:**
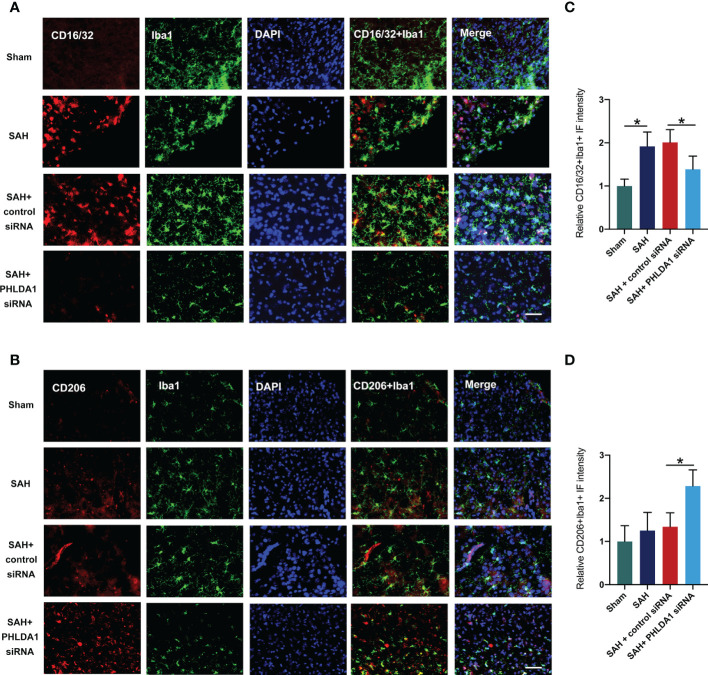
PHLDA1 deficiency inhibited M1 microglia polarization and promoted M2 microglia transformation. Representative immunofluorescence images of CD16/32 **(A)** and CD206 **(B)** co-localized with Iba1 in temporal cortex. Quantification of Iba1^+^/CD16/32^+^ cells **(C)** and Iba1^+^/CD206^+^ **(D)** cells in temporal cortex (n = 6 per group). ^*^P < 0.05. Scale bar=50 μm. Data are expressed as mean ± S.D.

Microglial polarization plays a key role in inflammatory response after SAH. Studies have proved that suppression of microglia M1 polarization and promotion of M2 microglia could effectively reduce acute brain injuries and improve neurological outcomes. Interestingly, PHLDA1 has been reported to modulate microglia M1/M2 polarization in other diseases. To determine whether PHLDA1 deficiency affects microglial polarization after SAH, double immunostaining was performed to examine the levels of M1 microglia and M2 microglia. It showed that the number of Iba1^+^/CD16/32^+^ cells was significantly increased after SAH, which could be decreased by PHLDA1 silencing (*P* < 0.05) (**Figures 4A, C**). In addition, we examined the expression of M2 microglia and revealed that the levels of Iba1^+^/CD206^+^ cells were markedly increased after PHLDA1 siRNA treatment (*P* < 0.05) (**Figures 4B, D**). These data suggested that PHLDA1 silencing could suppress M1 microglia and promote M2 microglia polarization.

### 3.5 PHLDA1 deficiency reduced neuronal death and improved neurological outcomes after SAH

**Figure 5:**
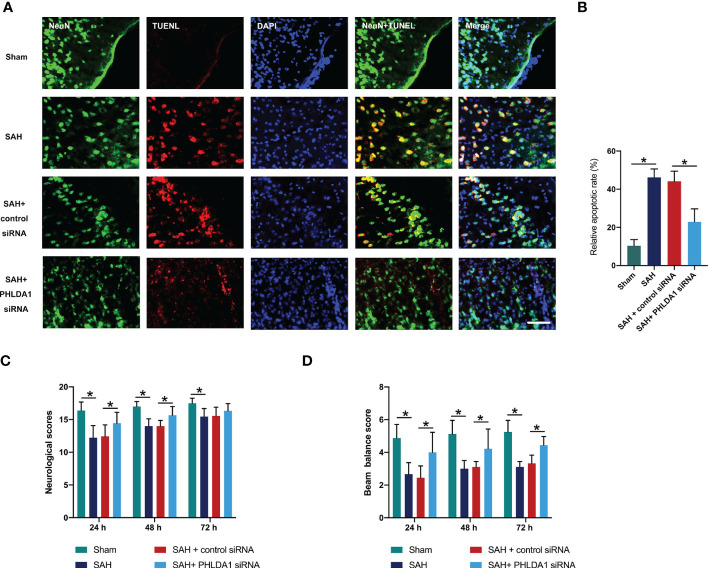
PHLDA1 deficiency decreased neuronal apoptosis and improved neurological function after SAH. **(A)** Representative immunofluorescence images of TUNEL staining in temporal cortex. **(B)** Quantification of TUNEL^+/^NeuN^+^ cells in temporal cortex (n = 6 per group). PHLDA1 deficiency mitigated neurological deficits **(C)** and improved motor function **(D)** after SAH (n = 8 or 9 per group). ^*^P < 0.05. Scale bar=50 μm. Data are expressed as mean ± S.D.

Next, we explored whether PHLDA1 deficiency could exert cerebroprotective effects after SAH. TUNEL staining revealed that SAH insults significantly induced neuronal apoptosis (*P* < 0.05) (**Figures 5A, B**). Concomitant with the exacerbated neuronal death, the neurological outcomes of SAH was further aggravated (*P* < 0.05) (**Figures 5C, D**). In contrast, PHLDA1 siRNA treatment evidently reduced SAH-induced neuronal apoptosis (*P* < 0.05). Simultaneously, PHLDA1 deficiency showed better neurological outcomes after SAH insults (*P* < 0.05) (**Figures 5A–D**). These suggested that PHLDA1 deficiency could protect against SAH-induced EBI by its anti-inflammatory effects.

### 3.6 Nigericin administration reversed the inhibitory effects of PHLDA1 deficiency on NLRP3 inflammasome

**Figure 6:**
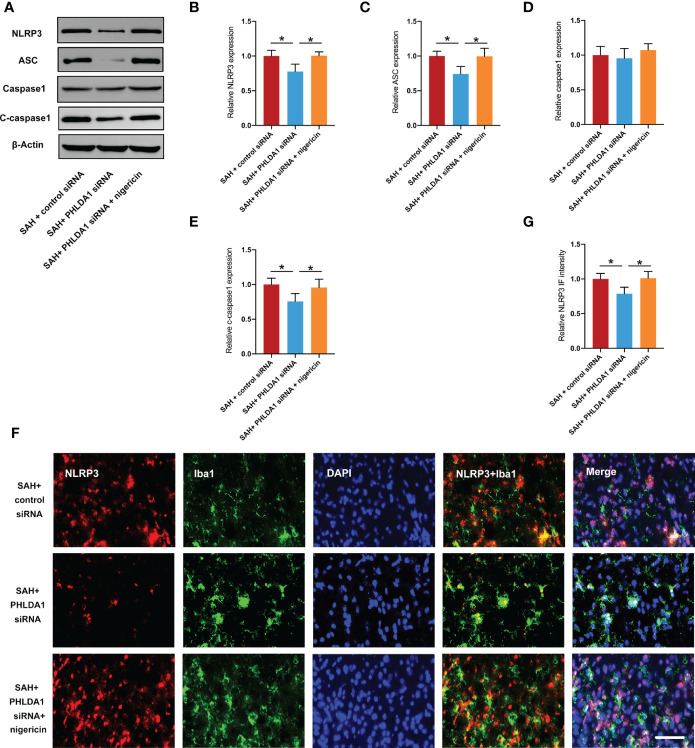
Nigericin administration counteracted the effects of PHLDA1 deficiency on NLRP3 inflammasome. **(A)** Representative western blots for NLRP3, ASC, caspase1, and cleaved caspasse1 expressions after SAH. Western blot analysis of NLRP3 **(B)**, ASC **(C)**, Caspase1 **(D)**, and Cleaved caspasse1 **(E)** expressions after SAH (n = 6 per group). **(F)** Representative immunofluorescence images of NLRP3 co-localized with Iba1 in temporal cortex. Quantification of NLRP3 **(G)** immunoactivity in microglia (n = 6 per group). ^*^P < 0.05. Scale bar=50 μm. Data are expressed as mean ± S.D.

NLRP3 inflammasome plays a key role in microglial activation after SAH. Moreover, NLRP3 inflammasome activation could induce microglia M1 polarization and inhibit NLRP3 inflammasome could promote M2 microglia polarization. As mentioned above, PHLDA1 deficiency could markedly inhibit NLRP3 inflammasome activation after SAH. We further explored whether NLRP3 inflammasome activation by nigericin could abate the cerebroprotective effects of PHLDA1 deficiency. As expected, western blot results showed that nigericin administration eliminated the inhibitory effects of PHLDA1 deficiency on NLRP3 inflammasome activation (*P* < 0.05) (**Figures 6A–E**). Consistently, the immunofluorescence staining results verified that NLRP3 inflammasome staining was further induced after nigericin treatment (*P* < 0.05) (**Figures 6F, G**).

### 3.7 Nigericin abated the effects of PHLDA1 deficiency on microglia M1/M2 polarization and inflammatory insults

**Figure 7:**
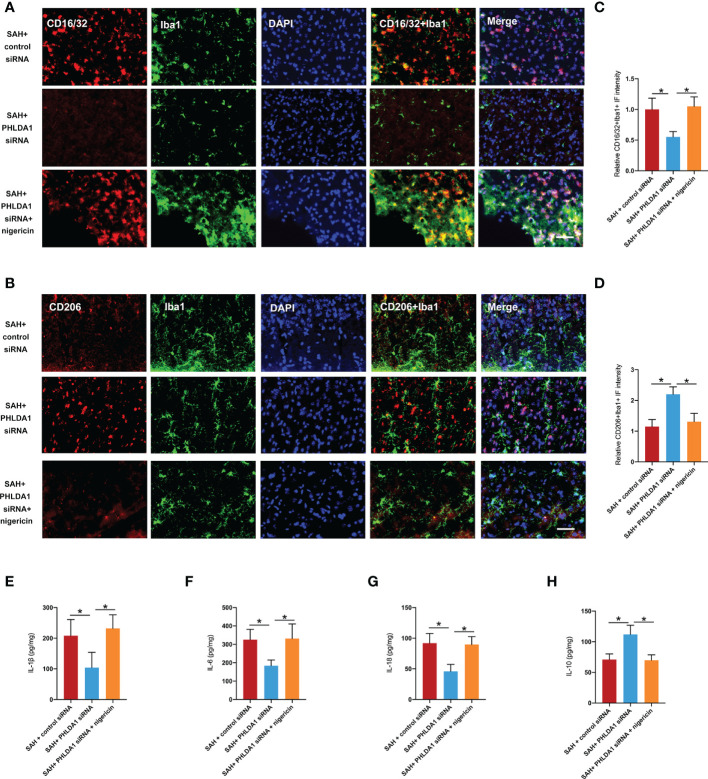
Nigericin administration abated the effects of PHLDA1 deficiency on microglia M1/M2 polarization and inflammatory insults. Representative immunofluorescence images of CD16/32 **(A)** and CD206 **(B)** co-localized with Iba1 in temporal cortex. Quantification of Iba1^+^/CD16/32^+^ cells **(C)** and Iba1^+^/CD206^+^ cells **(D)** in temporal cortex (n = 6 per group). ELISA analysis of IL-1β **(E)**, IL-6 **(F)**, IL-18 **(G)**, and IL-10 **(H)** expressions (n = 6 per group). ^*^P < 0.05. Scale bar=50 μm. Data are expressed as mean ± S.D.

Next, we examined the effects of nigericin administration on microglial polarization after PHLDA1 siRNA treatment. As expected, the immunofluorescence staining results showed that NLRP3 inflammasome activation by nigericin abated the effects of PHLDA1 silencing on microglia M1/M2 polarization, as evidenced by the increased number of Iba1^+^/CD16/32^+^ cells and decreased number of Iba1^+^/CD206^+^ cells (*P* < 0.05) (**Figures 7A–D**). Moreover, the ELISA data indicated that nigericin further exacerbated proinflammatory cytokines release and decreased anti-inflammatory cytokines after SAH (*P* < 0.05) (**Figures 7E–H**). These suggested that NLRP3 inflammasome activation contributed to the modulation effects of PHLDA1 on microglial polarization after SAH.

### 3.8 Nigericin abrogated the beneficial effects of PHLDA1 deficiency on neuronal survival and neurological function

**Figure 8:**
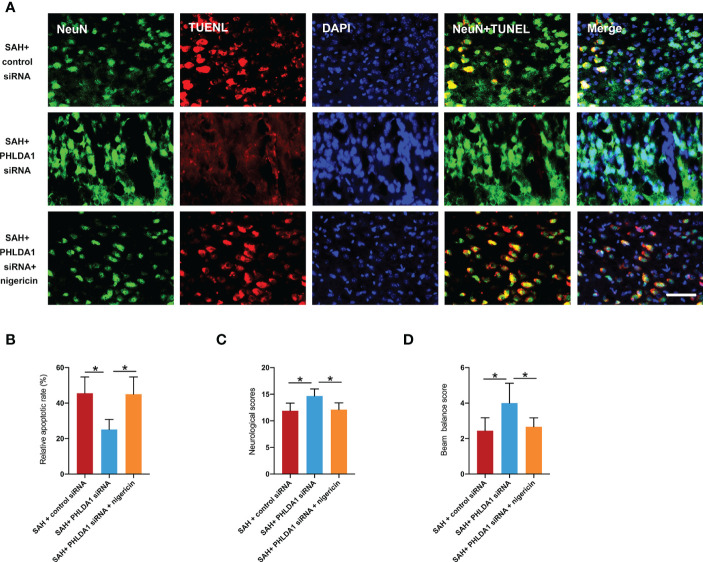
Nigericin administration abrogated the beneficial effects of PHLDA1 deficiency on neuronal survival and neurological function. **(A)** Representative immunofluorescence images of TUNEL staining in temporal cortex. **(B)** Quantification of TUNEL^+/^NeuN^+^ cells in temporal cortex (n = 6 per group). Nigericin administration aggravated neurological deficits **(C)** and motor dysfunction **(D)** after SAH (n = 9 per group). ^*^P < 0.05. Scale bar=50 μm. Data are expressed as mean ± S.D.

We suspected that NLRP3 inflammasome activation by nigericin might reverse the beneficial effects of PHLDA1 deficiency on neuronal survival and neurological function. Consistent with the aggravated inflammatory insults, nigericin administration further significantly increased neuronal apoptosis and exacerbated neurological deficits and motor dysfunction (*P* < 0.05) (**Figures 8A–D**). Based on the findings above, PHLDA1 blockade could attenuate EBI after SAH by regulating microglia M1/M2 polarization *via* NLRP3 inflammasome signaling.

## 4 Discussion

In this study, we elucidated the biological function of PHLDA1 in microglia-mediated neuroinflammation after SAH. We demonstrated that PHLDA1 was significantly increased and was peaked at 24 h after SAH. The immunofluorescence studies revealed that the enhanced PHLDA1 after SAH was mainly distributed in microglia. PHLDA1 siRNA treatment significantly reduced neuroinflammatory response and the subsequent brain insults after SAH. Notably, PHLDA1 knockdown reduced the number of M1 microglia and promoted M2 microglial polarization. Moreover, PHLDA1 deficiency inhibited NLRP3 inflammasome signaling after SAH. In contrast, NLRP3 inflammasome activator nigericin abrogated the protective effects of PHLDA1 deficiency against SAH and further aggravated neurobehavior deficits. Taken together, these data suggested that targeting PHLDA1 might be a potential therapeutic strategy for treating SAH.

PHLDA1, a member of the PHLDA family, is a multifunctional protein. It has been demonstrated that PHLDA1 participates in modulation of cell proliferation, energy homeostasis, differentiation and apoptosis (27–29). Recently, a wealth of evidence indicated that PHLDA1 also plays an important role in immune response. For example, Hossain et al. demonstrated that PHLDA1 knockdown modulated macrophages and endothelial cells phenotypic changes to reduce atherogenesis-induced oxidative and ER stress (14). In Parkinson’s disease study, Han et al. reported that PHLDA1 was a potent modulator of neuroinflammation, and knockdown of PHLDA1 could markedly inhibited M1 microglia activation (13). A more direct study indicated that PHLDA1 blockade inhibited neuroinflammation after ischemic stroke by balancing microglial M1/M2 polarization (12). However, the potential roles of PHLDA1 in EBI after SAH remain unclear.

We first investigated the time course of PHLDA1 expression after SAH. Consistent with previous reports (12), our data indicated that PHLDA1 was significantly increased, with a peak at 24 h after SAH. At the cellular level, the enhanced PHLDA1 after SAH was mainly distributed in microglia. Moreover, we noted that NLRP3 inflammasome was significantly increased in microglia after SAH. The NLRP3 inflammasome, a cytoplasmic multiprotein complex, has been demonstrated to exert a pivotal role in neuroinflammation and intracranial aneurysm rupture (30–32). It showed that NLRP3 inflammasome participated in microglial polarization in a variety of brain injuries (33–35). Chen et al. demonstrated that NLRP3 deficiency could promote M2 microglia polarization in experimental models of intracerebral hemorrhage (36). A recent study in cerebral ischemia/reperfusion injury suggested that inhibition of TXNIP/NLRP3 promoted the transition of microglia from M1 to M2 phenotype (37). In SAH area, inhibition NLRP3 inflammasome has also been demonstrated to promote microglial polarization to M2 phenotype (4). In our experiments, we observed that there were similar expression trends and cellular distribution of PHLDA1 and NLRP3 after SAH. These suggested that PHLDA1 and NLRP3 might interact with each other. Intriguingly, a recent study by Zhao et al. revealed that PHLDA1 deficiency suppressed middle cerebral artery occlusion/reperfusion-induced NLRP3 inflammasome activation and the subsequent mRNA level and expression of NLRP3 inflammasome-associated proteins (12). Therefore, we speculated that PHLDA1 might modulate microglial polarization by NLRP3 inflammasome activation.

In our experiments, we employed PHLDA1 siRNA to suppress PHLDA1 activation. Our data revealed that PHLDA1 siRNA treatment significantly inhibited the protein expression of PHLDA1 after SAH. Moreover, the evident neuroinflammation was markedly suppressed by PHLDA1 blockade. Microglial polarization plays a critical role in immune response after SAH. Mounting evidence has shown that inducing microglia toward M2 phenotype or suppression of microglia M1 polarization could promote neuronal survival, reduce inflammatory insults, and improve neurological outcomes after SAH (38–40). We further examined the influence of PHLDA1 blockade on microglial polarization after SAH. Consistent with previous studies, we found that PHLDA1 deficiency decreased M1 phenotype microglia and induced microglia M2 polarization after SAH. Concomitant with the reduced neuroinflammation, PHLDA1 blockade improved neuronal survival and neurological function after SAH. However, the underlying molecular mechanisms of PHLDA1 blockade on microglial polarization remain unknown. The evidence above implied that NLRP3 inflammasome might participate in PHLDA1-mediated microglial polarization. To further clarify the molecular mechanisms of PHLDA1 blockade on microglial polarization, nigericin was applied to activate NLRP3 inflammasome signaling. As expected, nigericin treatment significantly induced NLRP3 inflammasome activation and abrogated the beneficial effects of PHLDA1 blockade on EBI after SAH. Meanwhile, nigericin further increased M1 microglia polarization and inhibited M2 phenotype microglia. These data further supported that PHLDA1 blockade could inhibit NLRP3 inflammasome activation to balance microglial polarization from M1 to M2 after SAH.

There are several shortcomings in our study. Firstly, microglia-specific PHLDA1-knockout mouse should be utilized in the future to validate the biological function of PHLDA1 in microglial activation after SAH. Secondly, some authors reported that PHLDA1 exhibited anti-inflammatory effects through inhibition of toll-Like receptor 4 (TLR4) signaling (15). The reasons for this disagreement are somewhat obscure. One possible explanation might be that acute brain injuries have different pathophysiology. Thirdly, in addition to modulate NLRP3 inflammasome signaling, PHLDA1 might interfere with other molecular targets, such as TLR4, nuclear factor-erythroid 2-related factor 2, and TRAF6 (13, 15, 41). Moreover, the long-term effects of PHLDA1 inhibition in the delayed phase of SAH remains unclear. Therefore, additional studies are still warranted to clarify these questions.

## 5 Conclusions

This study is the first to document that PHLDA1 blockade attenuated EBI after SAH by regulating microglia M1/M2 polarization *via* NLRP3 inflammasome signaling. These findings suggested that PHLDA1 might be a novel therapeutic strategy for treating SAH.

## Data availability statement

The original contributions presented in the study are included in the article/supplementary material. Further inquiries can be directed to the corresponding authors.

## Ethics statement

The animal study was reviewed and approved by the Animal Core Facility of Fujian Medical University.

## Author contributions

JL and XC conceived and designed the experiments; JL, GC, ZW, SY, RH, and YZ performed research; WL conducted the data analysis; JL, CF, and XC revised the manuscript. All authors contributed to the article and approved the submitted version.
